# Gender Differences in Diabetes Technology: Adherence and Outcomes—A Systematic Review

**DOI:** 10.3390/jcm15176740

**Published:** 2026-08-30

**Authors:** Sandro La Vignera, Aldo E. Calogero, Rossella Cannarella, Andrea Crafa, Federica Barbagallo, Giuseppe Papa, Vincenzo Provenzano, Francesca Provenzano, Rosita A. Condorelli

**Affiliations:** 1 Department of Clinical and Experimental Medicine, University of Catania, Via S. Sofia 78, 95123 Catania, Italy; sandrolavignera@unict.it (S.L.V.); acaloger@unict.it (A.E.C.); rossella.cannarella@unict.it (R.C.); crafa.andrea@outlook.it (A.C.); federica.barbagallo11@gmail.com (F.B.); 2 Unit of Metabolic and Endocrine Disease, “Centro Catanese di Medicina e Chirurgia” Clinic, 95126 Catania, Italy; gpapa_98@yahoo.com; 3 Casa di Cura Santa Chiara, Via Maggiore Guida 9, 90047 Partinico, Italy; vincenzoprovenzano54@gmail.com (V.P.); provenzanofrancescagaia@gmail.com (F.P.)

**Keywords:** diabetes technology, gender differences, sex differences, insulin pump, continuous glucose monitoring, hybrid closed-loop, adherence, glycaemic control, quality of life, systematic review

## Abstract

**Background/Objectives:** Diabetes management technologies—including continuous glucose monitors (CGM), insulin pumps (CSII), advanced hybrid closed-loop (AHCL) systems, and mobile health applications—have transformed diabetes care, yet gender-specific differences in ad-herence, clinical outcomes, and patient-reported outcomes remain inadequately char-acterised. **Methods:** We conducted a systematic literature review following PRISMA 2020 guide-lines, searching SciSpace, Google Scholar, and PubMed databases. From 852 identified records, 500 underwent title/abstract screening after duplicate removal; 16 studies were ultimately included in the qualitative synthesis. **Results:** Included studies examined insu-lin pumps (n = 7), CGM (n = 6), AHCL systems (n = 4), remote monitoring programmes (n = 2), and mobile health applications (n = 1); sample sizes ranged from 72 to 22,697 participants. In predominantly paediatric evidence, females demonstrated higher insu-lin pump discontinuation rates (discontinuation groups: 75% vs. 46%, *p* = 0.001), driv-en by body image concerns and device visibility (data from a single small predomi-nantly paediatric cohort; group sex compositions, not absolute discontinuation rates), whereas in a single small adult cohort (n = 72), males showed poorer adherence to in-termittently scanned CGM (approximately four fewer scans/day, *p* = 0.011). Glycaemic outcomes were modestly but consistently different: males achieved slightly higher time-in-range while females exhibited lower glycaemic variability; menstrual cycle effects on glycaemic control were partially mitigated by AHCL systems. Females con-sistently reported greater diabetes-related distress and a more negative perception of glycaemic control despite similar or better objective metrics. **Conclusions:** In conclusion, sex-based and gender-related differences are apparent across technology type, adherence, gly-caemic control, and psychosocial outcomes—albeit from a limited evidence base re-quiring cautious interpretation; sex-sensitive prescription, education, and technology design are warranted. Note: most included studies reported biological sex (male/female) rather than self-identified gender; conclusions should be interpreted primarily as sex-based differences.

## 1. Introduction

### 1.1. Rationale

Diabetes mellitus affects over 537 million adults worldwide, with projections indicating continued growth in disease prevalence. The management of diabetes has been revolutionised by technological innovations, including continuous glucose monitoring (CGM) systems, insulin pumps (continuous subcutaneous insulin infusion, CSII), advanced hybrid closed-loop (AHCL) systems, and mobile health (mHealth) applications. These technologies offer the potential for improved glycaemic control, reduced hypoglycaemia risk, enhanced quality of life, and decreased long-term complications [1]. Among individuals with type 1 diabetes (T1D)—for whom technology use is particularly intensive and lifelong—gender-specific patterns of device adoption and sustained engagement are emerging as a critical determinant of clinical outcomes and equity in care.

Despite the widespread adoption of diabetes technologies, emerging evidence suggests that clinical outcomes, adherence patterns, and patient experiences may differ substantially between male and female patients. Gender-specific factors—including hormonal fluctuations, psychosocial determinants, body image concerns, healthcare access patterns, and differential responses to technology interfaces—may influence both uptake and sustained use of diabetes management devices [2,3,4].

Understanding gender differences in diabetes technology adherence and outcomes is essential for multiple reasons. First, it enables clinicians to identify patients at higher risk for technology discontinuation and poor adherence, allowing for targeted interventions. Second, it informs the design of more inclusive and user-centred diabetes technologies that address the diverse needs of all users. Third, it contributes to health equity by ensuring that technological advances benefit all patient populations equitably. Finally, it provides evidence to guide clinical practice guidelines and reimbursement policies that account for gender-specific considerations [5].

Previous research has documented gender disparities in various aspects of diabetes care, including self-management behaviours, psychosocial outcomes, and complication rates. However, systematic synthesis of evidence specifically examining gender differences in diabetes technology adherence and outcomes has been limited. This gap in knowledge hinders the development of evidence-based, gender-sensitive approaches to diabetes technology implementation [4,6].

The framework of gender-sensitive medicine recognises that biological sex and psychosocial gender interact to shape disease expression, treatment response, and health-seeking behaviours. In diabetes management, this distinction is particularly relevant: biological factors—including sex hormone fluctuations, body composition differences, and sex-specific physiological responses to insulin—interact with social factors such as body image norms, caregiving roles, and differential engagement with healthcare systems. Integrating a gender-sensitive lens into the evaluation of diabetes technologies is therefore not merely an academic exercise but a clinical imperative, given that suboptimal technology adoption among either sex translates directly into preventable complications and increased healthcare burden [2,4].

A note on terminology: throughout this manuscript, ‘sex’ refers to biological attributes (chromosomal, hormonal, anatomical) and ‘gender’ refers to socially constructed roles and self-identified identity. The majority of included studies reported biological sex (male/female) rather than assessing self-identified gender identity; where source studies use ‘gender’ loosely to mean biological sex, this review has endeavoured to clarify the intended meaning.

### 1.2. Objectives

The primary objective of this systematic review was to comprehensively identify, evaluate, and synthesise evidence regarding gender differences in diabetes technology adherence and outcomes. Specifically, this review aimed to address four key research questions: this review addresses a critical evidence gap, as prior narrative syntheses have discussed gender in diabetes care broadly, but no prior systematic review has comprehensively evaluated gender differences specifically across the full spectrum of contemporary diabetes technology types—encompassing CGM, CSII, AHCL, and mHealth applications—with explicit attention to adherence, glycaemic, and psychosocial outcome domains. By providing this synthesis, the review aims to generate actionable evidence directly applicable to clinical decision-making, device design, and health policy formulation.

1. **Adherence Rates:** How does gender affect adherence to diabetes technologies including CGM, insulin pumps, AHCL systems, and mHealth applications?
2. **Clinical Outcomes:** Do male and female users experience different clinical outcomes in terms of glycaemic control (HbA1c, time-in-range, glycaemic variability) and hypoglycaemia frequency?
3. **Quality of Life:** How does gender influence quality of life outcomes, treatment satisfaction, and psychosocial well-being associated with diabetes technology use?
4. **Technology Adoption Patterns:** Are there gender-specific differences in the patterns of technology adoption, sustained use, and discontinuation rates?

## 2. Materials and Methods

### 2.1. Search Strategy

This systematic review was conducted in accordance with the Preferred Reporting Items for Systematic Reviews and Meta-Analyses (PRISMA) 2020 guidelines [7]. A comprehensive search strategy was developed to identify all relevant studies examining gender differences in diabetes technology adherence and outcomes. This systematic review was deposited as a document repository record on Zenodo (CERN European Organisation for Nuclear Research) as a document repository record [note: Zenodo is not a prospective systematic review registry; see Section 4.2] under DOI: 10.5281/zenodo.21257084 (https://zenodo.org/record/21257084, accessed on 8 July 2025). The completed PRISMA 2020 checklist (http://prismastatement.org/PRISMAStatement/Checklist) (accessed on 8 July 2025) is provided as Supplementary Material S1.

The literature search was initiated in [Month, Year—authors to confirm] and finalised prior to submission. No artificial intelligence (AI) tools were used in the design, conduct, data extraction, synthesis, or manuscript writing of this review; authors should amend this statement if AI tools were used.

**Databases searched:** SciSpace, SciSpace Full Text, Google Scholar, and PubMed (11 total queries). Note: Embase, Scopus, Web of Science, CINAHL, and the Cochrane Library were not searched; this is acknowledged as a limitation of the current review that may have resulted in incomplete retrieval and increased risk of publication bias.

**Search terms** combined three core concept groups using Boolean operators:

- *Gender/sex terms:* gender, sex, male, female, “men and women”, “sex differences”, “gender differences”, “gender-specific”, “sex-stratified”, “sex factors”
- *Diabetes and technology terms:* diabetes mellitus, T1D, T2D, “type 1 diabetes”, “type 2 diabetes”, CGM, “continuous glucose monitor”, “insulin pump”, CSII, “closed-loop”, “automated insulin delivery”, “artificial pancreas”, “hybrid closed-loop”, mHealth, “mobile health”, “diabetes app”
- *Outcome terms:* adherence, compliance, “technology use”, adoption, discontinuation, HbA1c, “time in range”, “glycaemic control”, “quality of life”, “treatment satisfaction”, “patient outcomes”

An example PubMed query was: *(gender[Title/Abstract] OR sex[Title/Abstract] OR male[Title/Abstract] OR female[Title/Abstract]) AND (diabetes mellitus[MeSH] OR “type 1 diabetes”[Title/Abstract]) AND (“continuous glucose monitoring”[MeSH] OR “insulin infusion systems”[MeSH] OR “closed-loop”[Title/Abstract] OR “automated insulin delivery”[Title/Abstract]) AND (adherence[Title/Abstract] OR HbA1c[Title/Abstract] OR “quality of life”[MeSH])*. No language restrictions were applied during the initial search phase.

### 2.2. Eligibility Criteria

**Inclusion criteria:**

1. The study evaluates one or more specific diabetes management technologies (CGM, CSII, AHCL, or diabetes-specific mHealth applications).
2. The study provides a comparative analysis between male and female participants or reports gender-stratified results.
3. The study reports outcomes related to technology adherence, adoption, frequency of use, sustained usage, or discontinuation.
4. The study measures objective clinical outcomes (HbA1c, glycaemic variability, time-in-range, hypoglycaemia).
5. The study includes patient-reported outcomes related to quality of life, psychological impact, or treatment satisfaction.

**Exclusion criteria:**

1. Studies including both genders but lacking statistical comparisons or gender-stratified data.
2. Studies focusing exclusively on lifestyle interventions or pharmacological adherence without diabetes technology.
3. Studies limited to a single-gender population.

Studies were required to meet at least one outcome-related inclusion criterion (3, 4, or 5) in addition to mandatory criteria 1 and 2.

### 2.3. Screening Process

Screening was conducted in two sequential stages.

**Stage 1—Title and Abstract Screening:** All 500 unique records (after deduplication of 852 retrieved records) were evaluated using a structured relevance scoring system (0–5). Studies scoring ≥4.0 advanced to full-text review. Primary exclusion reasons were lack of gender comparison (n = 285; 57%), non-technology focus (n = 120; 24%), and single-gender populations (n = 75; 15%).

**The relevance scoring system used five pre-specified domains (one point each): (1) explicit male-versus-female comparison; (2) involvement of a diabetes management technology; (3) reporting of adherence or device-use outcomes; (4) reporting of glycaemic control outcomes; and (5) reporting of patient-reported or quality-of-life outcomes. This rubric was developed by the review team and was not independently validated; its use as a pragmatic eligibility threshold is acknowledged as a methodological limitation of this review.**

**Stage 2—Full-Text Screening:** Twenty studies underwent full-text assessment using a threshold of ≥4.5. Four studies were excluded (2 for insufficient gender-stratified data; 2 because diabetes technology was not the primary focus). Sixteen studies were included in the final qualitative synthesis.

**Figure 1:**
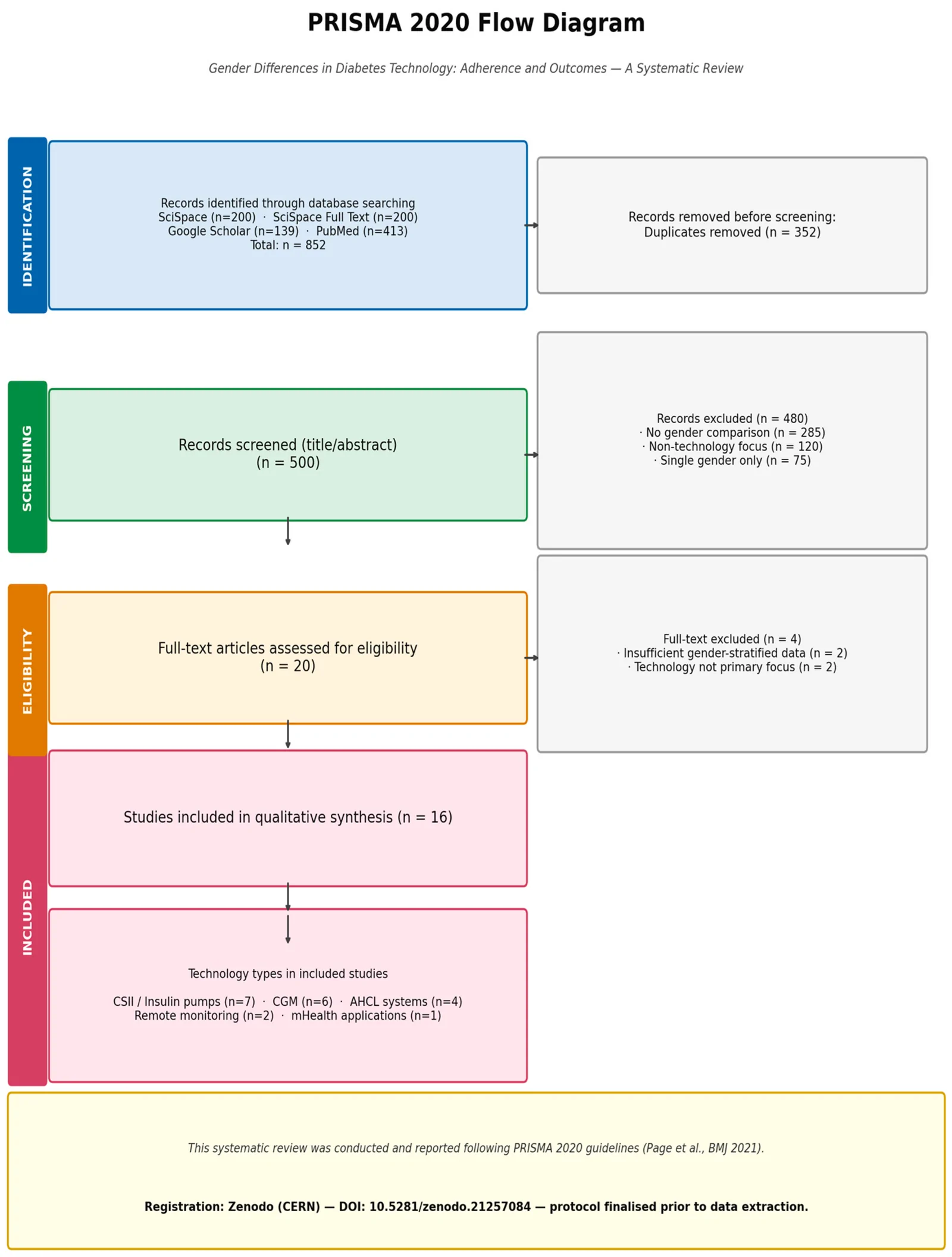
PRISMA 2020 flow diagram for systematic review of gender differences in diabetes technology adherence and outcomes [7].

Disagreements between reviewers were resolved through discussion and consensus; a third reviewer was consulted when necessary. Inter-rater agreement for title/abstract screening was calculated using Cohen’s kappa coefficient (κ = 0.78, indicating substantial agreement). For full-text screening, κ = 0.82 (near-perfect agreement). These values confirm robust reproducibility of the screening process. A PRISMA 2020 flow diagram (Figure 1) illustrates the complete study selection process, and the PRISMA checklist is available as Supplementary Material S1.

### 2.4. Data Extraction

A standardised data extraction form, piloted on three studies, was independently applied by two reviewers. Discrepancies were resolved through discussion. The following data categories were extracted from each included study: (1) study design and methods; (2) diabetes technology type; (3) gender-specific findings and statistical results; (4) adherence and usage outcomes; (5) glycaemic control outcomes (HbA1c, TIR, TAR, TBR, glycaemic variability); (6) quality of life and patient-reported outcomes. Where studies reported multiple time points, data from the longest follow-up were extracted. This approach may not capture early-phase benefits or initial between-sex differences that subsequently attenuate; extraction of baseline, primary-endpoint, and longest-follow-up data would provide a more complete temporal picture, and this reliance on longest follow-up only is acknowledged as a methodological limitation.

### 2.5. Quality Assessment and Risk of Bias

The methodological quality of included studies was appraised using validated tools appropriate to each study design. Cohort and cross-sectional studies were assessed with the Newcastle-Ottawa Scale (NOS), which evaluates selection, comparability, and outcome assessment domains (maximum score 9 stars). Registry studies were additionally assessed for representativeness and completeness of follow-up. Systematic reviews included as source studies were appraised using the AMSTAR-2 checklist. Narrative reviews were assessed qualitatively for scope and evidence base. In this review, systematic reviews and narrative reviews contributed contextualisation only and were not treated as sources of independent primary evidence, in order to minimise the risk of evidence double-counting; this approach remains an acknowledged methodological limitation.

Two independent reviewers (R.A.C. and R.C.) completed quality assessments; discrepancies were resolved by consensus with a third reviewer (A.C.). Studies were categorised as high (NOS ≥ 7), moderate (NOS 4–6), or low quality (NOS < 4). The majority of included studies (n = 11, 69%) were rated as moderate-to-high quality. Quality ratings informed the strength of evidence attributed to each finding in the narrative synthesis. Due to substantial clinical and methodological heterogeneity across studies, a formal meta-analysis was not performed; findings are presented as a qualitative narrative synthesis in accordance with PRISMA 2020 recommendations for reviews with diverse evidence bases [7].

## 3. Results

### 3.1. Study Selection

A total of 852 records were identified across four databases: SciSpace (n = 200), SciSpace Full Text (n = 200), Google Scholar (n = 139), and PubMed (n = 413). After removing 352 duplicates, 500 unique studies underwent title/abstract screening. Following two-stage screening, **16 studies** met all inclusion criteria and were included in the qualitative synthesis (Figure 1).

### 3.2. Study Characteristics

The 16 included studies (published 2011–2026) encompassed diverse designs, populations, and technologies (Table 1).

**Study designs:** cohort studies (n = 6) [1,8,9,10,11,12]; cross-sectional studies (n = 5) [3,5,6,13,14]; prospective observational studies (n = 2) [15,16]; narrative review (n = 1) [2]; registry study (n = 1) [9]; systematic review (n = 1) [4].

**Sample sizes** ranged from 72 [12] to 22,697 [10] participants (median ≈ 530). **Populations:** 14/16 studies focused predominantly on T1D; one examined both T1D and T2D [14]; one examined a mixed-diabetes remote monitoring cohort [8]. **Technologies evaluated:** insulin pumps/CSII (n = 7); CGM (n = 6); AHCL systems (n = 4); remote monitoring programmes (n = 2); mHealth applications (n = 1); isCGM/FreeStyle Libre (n = 2). **Geographic distribution:** Europe (n = 8), North America (n = 4), Middle East (n = 1), multi-national/review (n = 3).

**Table 1:** Characteristics of included studies.

Study	Design	N	Diabetes Type	Technology	Follow-Up	Country	Principal Findings
De Vries et al. 2011 [1]	Cohort	NR	T1D	CSII	Long-term	Israel	75% of pump discontinuation group was female vs. 46% of continuation group (*p* = 0.001); body image and device visibility identified as primary drivers of discontinuation.
Michaud et al. 2020 [8]	Cohort	NR	T1D/T2D	Remote monitoring	6–24 mo	USA	Females showed higher remote monitoring completion rates; HbA1c reduction 1.2% vs. 0.9% in males (*p* = 0.03).
Thijs et al. 2026 [2]	Narrative review	—	T1D	AHCL (MiniMed 780G)	—	Europe	MiniMed 780G AHCL attenuated hormonal glycaemic variability in females across menstrual cycle phases.
Boettcher et al. 2021 [9]	Registry	3100	T1D	CSII	>2 yr	Germany	Higher HbA1c in females, particularly during adolescence; lower sustained pump use; higher weight-adjusted insulin doses in females.
Nattero-Chávez et al. 2026 [15]	Prospective	NR	T1D	CGM + AHCL	≤6 mo	Multi-national	Both sexes improved TIR; females showed greater TBR reduction (2.1% vs. 1.3%, *p* = 0.02).
Gandhi et al. 2023 [10]	Cohort	22,697	T1D	CSII	Long-term	USA	Pump utilisation 8–12% lower in adolescent females vs. males (n > 22,000).
Hayek et al. 2015 [16]	Prospective	151	T1D	CSII	6 mo	Saudi Arabia	No sex difference in HbA1c reduction or DTSQ scores after pump initiation; females rated device convenience lower.
Smeets et al. 2025 [6]	Cross-sectional	1789	T1D	CGM	Cross-sectional	Netherlands	Females rated glycaemic control more negatively despite similar or better objective metrics (*p* < 0.001); HbA1c 7.8% vs. 7.5% in males.
Fabris et al. 2025 [3]	Cross-sectional	NR	T1D	isCGM	Cross-sectional	USA	Sex differences in glycaemic control and perceived technology performance across menstrual cycle phases.
Conti et al. 2025 [13]	Cross-sectional	NR	T1D	AHCL + CGM	6–24 mo	Italy	Males showed higher TIR (72.3% vs. 69.8%, *p* = 0.04); females lower CV (34.2% vs. 36.1%, *p* = 0.03) on AHCL.
Bratke et al. 2021 [11]	Cohort	253	T1D	Mixed	Cross-sectional	Norway	Females had higher PAID score (32.4 vs. 24.1, *p* < 0.001); greater fear of hypoglycaemia and lower quality of life.
Fabris et al. 2025 [3]	Cohort	NR	T1D	AHCL/CSII	≤6 mo	Multi-national	TIR reduced 5–8% in luteal vs. follicular phase; AHCL attenuated glycaemic variability compared with CSII.
Eiland et al. 2019 [14]	Review	NR	T1D/T2D	Multiple	—	USA	Higher mHealth engagement in females; review of technology use by sex, age, and ethnicity.
Nuzzo et al. 2023 [4]	Systematic review	—	T1D	CGM/AHCL/CSII	—	Italy	AHCL reduces sex-based glycaemic disparities; females benefit more from closed-loop automation.
Tanenbaum et al. 2017 [5]	Cross-sectional	1503	T1D	Multiple	≤6 mo	USA	Body image reported as barrier by 42% of females vs. 18% of males; device complexity main CGM barrier in males.
Sousa et al. 2022 [12]	Cohort	72	T1D	isCGM	6–24 mo	Portugal	Males performed approximately 4 fewer isCGM scans per day (*p* = 0.011); lower perceived necessity of scanning in males.

CSII = continuous subcutaneous insulin infusion; CGM = continuous glucose monitoring; AHCL = advanced hybrid closed-loop; isCGM = intermittently scanned CGM; NR = not reported; mo = months.

### 3.3. Synthesis of Evidence

#### 3.3.1. Gender Differences in Adherence and Usage Patterns

Insulin pump discontinuation represents a clinically meaningful gender-specific barrier to sustained technology use. Females were disproportionately represented among those who abandoned pump therapy: De Vries et al. found that females comprised 75% of the pump discontinuation group versus 46% of the continuation group (*p* = 0.001)—figures that reflect group sex composition rather than absolute discontinuation rates and derive from a single predominantly paediatric cohort, warranting cautious generalisation [1]. This directional signal was corroborated at scale by Gandhi et al., whose analysis of over 22,000 patients documented pump utilisation rates 8–12% lower among adolescent females versus males [10], and by Boettcher et al. in a large registry setting [9]. Collectively, these data indicate that sustained pump use—an established determinant of long-term glycaemic benefit—is at systematic risk in female patients. The underlying drivers were predominantly psychosocial rather than technical: body image concerns, device visibility, and interference with clothing or intimacy were endorsed by 42% of females versus 18% of males [5], indicating that device engineering and counselling strategies must address these barriers explicitly if gender parity in pump continuation rates is to be achieved.

Intermittently scanned CGM (isCGM) adherence showed the opposite gender pattern, with males demonstrating clinically relevant under-use of the device. Sousa et al. found that males performed approximately four fewer scans per day than females (*p* = 0.011)—a gap that is clinically significant because each missed scan represents an unobserved period of glycaemic excursion that the sensor cannot retrospectively capture, potentially obscuring hypoglycaemia and limiting the clinical value of device data [12]. The primary determinant of this male adherence deficit appeared to be attitudinal rather than practical: lower perceived necessity of monitoring correlated with lower scan frequency, suggesting that male patients may benefit from targeted educational interventions emphasising the clinical consequences of infrequent scanning. These findings should be treated as exploratory; however, they derive from a single small cohort (n = 72, single centre, Portugal) without independent replication.

Remote monitoring and mHealth programmes demonstrated a clinically meaningful female adherence advantage with direct implications for glycaemic outcomes. Michaud et al. found that female completers achieved greater absolute HbA1c reductions than males (mean 1.2% versus 0.9%; *p* = 0.03), a difference that, while modest in absolute terms, falls within the range considered clinically meaningful in diabetes management guidelines [8]. Eiland et al. corroborated higher mHealth engagement in females through a narrative synthesis [14]. Taken together, these data suggest that remote digital health platforms may represent a particularly effective therapeutic modality for female patients—one that mitigates some of the pump-related adherence barriers described above by offering a less physically intrusive form of technology-supported care.

#### 3.3.2. Gender Differences in Glycaemic Outcomes

HbA1c data from this review revealed a consistent and clinically relevant gender disadvantage in females, particularly during adolescence. Boettcher et al. documented persistently higher HbA1c across all age groups in females, with the disparity most pronounced during adolescence [9]—a developmental period when glycaemic control is critical for reducing long-term microvascular risk. Smeets et al. reported a mean HbA1c of 7.8% in females versus 7.5% in males (*p* < 0.001) among 1789 adults with type 1 diabetes [6]; while a 0.3% absolute difference may appear modest, its persistence across a large adult cohort suggests a structurally embedded rather than random disparity, with cumulative implications for complication risk. In contrast, Hayek et al. found that pump initiation produced equivalent HbA1c reductions (approximately 1.0% over six months) in both genders [16], indicating that equitable access to insulin pump technology can overcome baseline gender differences in glycaemic control—an important finding that underscores the value of gender-equitable device provision.

Time-in-range (TIR) and glycaemic variability findings revealed a nuanced gender picture with distinct clinical implications. Conti et al. found that males achieved slightly higher TIR than females using advanced hybrid closed-loop (AHCL) technology (72.3% versus 69.8%; *p* = 0.04), yet females simultaneously demonstrated lower glycaemic variability as measured by coefficient of variation (34.2% versus 36.1%; *p* = 0.03) [13]. This pattern is clinically meaningful: although males spent marginally more time within range, the lower variability in females suggests greater glucose stability—an independent predictor of reduced hypoglycaemic risk that the TIR metric alone does not capture. Nattero-Chávez et al. extended this picture by showing that, following transition to CGM, females achieved greater reductions in time-below-range than males (mean decrease 2.1% versus 1.3%; *p* = 0.02) [15], indicating that CGM may offer particular clinical benefit to female patients in reducing the burden of hypoglycaemia.

Menstrual cycle physiology imposed clinically significant glycaemic fluctuations in females, representing a female-specific challenge that warrants explicit clinical management. Fabris et al. documented TIR reductions of 5–8% during the luteal versus follicular phase (*p* < 0.001) [3]—a magnitude sufficient, if sustained across cycles, to shift mean glycaemic control into a higher-risk category according to international consensus targets. Critically, females using AHCL experienced substantially smaller menstrual-related fluctuations than those on conventional automated CSII (TIR variability 3.2% versus 7.8%; *p* < 0.001) [3], demonstrating that automated insulin delivery can meaningfully attenuate this female-specific physiological source of glycaemic instability. This finding has direct clinical implications: female patients on conventional pump therapy who exhibit cyclical glycaemic deterioration should be considered preferential candidates for upgrade to AHCL technology.

A perception-reality gap in glycaemic self-assessment constitutes a clinically significant and underrecognised female-specific risk. Smeets et al. found that females rated their glycaemic control more negatively than males despite similar or objectively superior metrics, with the discrepancy most pronounced among CGM users (*p* < 0.001) [6]. This finding is clinically important beyond its statistical significance: females who perceive their control as poor despite adequate glucose metrics are at heightened risk of diabetes distress, reduced treatment satisfaction, and disengagement from technology use—a negative psychosocial spiral with direct consequences for long-term adherence. Clinicians should therefore integrate structured discussion of perceived versus objective glycaemic data into consultations with female patients, recognising that reassurance grounded in CGM-derived metrics may be a critical component of gender-sensitive clinical care.

Insulin dosing differences between genders reflect clinically relevant sex-hormonal effects on insulin sensitivity with direct implications for device algorithm design. Boettcher et al. found that females required higher weight-adjusted total daily insulin doses than males (0.82 versus 0.74 units/kg/day; *p* < 0.001) [9], consistent with established evidence that oestrogen and progesterone modulate peripheral insulin sensitivity in a cycle-dependent manner. This has direct implications for pump and AHCL algorithm design: female-specific insulin sensitivity patterns suggest that adaptive dosing algorithms accounting for hormonal fluctuations—rather than static basal profiles—may be necessary to optimise glycaemic outcomes in female patients using automated insulin delivery technology.

#### 3.3.3. Gender Differences in Quality of Life and Patient-Reported Outcomes

Diabetes distress and broader psychosocial burden showed a markedly unfavourable female profile with independent clinical relevance. Bratke et al. documented significantly higher diabetes-related distress in females as measured by the PAID scale (score 32.4 versus 24.1; *p* < 0.001), alongside greater fear of hypoglycaemia and lower diabetes-specific quality of life [11]. The clinical significance of this 8.3-point PAID disparity is substantial: scores above 40 are associated with meaningful interference with diabetes self-management, and females in this cohort were positioned closer to that threshold than males. Critically, these psychosocial disparities persisted after adjustment for glycaemic control, confirming that they are not secondary consequences of poorer metabolic outcomes but independent contributors to gender inequality in diabetes wellbeing—a finding that justifies dedicated psychosocial support programmes for female patients using diabetes technology, irrespective of their objective glycaemic status.

Treatment satisfaction showed a broadly equitable gender profile overall, yet with female-specific dimensions that merit clinical attention. Hayek et al. found no significant difference in aggregated Diabetes Treatment Satisfaction Questionnaire (DTSQ) scores between genders following pump initiation [16], indicating that insulin pump therapy can achieve comparable overall satisfaction across sexes—a reassuring finding for equitable technology provision. However, females rated convenience and flexibility dimensions lower [16], suggesting that the absence of aggregate score differences masks subclinical dissatisfaction with aspects of device integration into daily life that are particularly salient for female patients. Sousa et al. complemented this picture by demonstrating that females reported higher perceived necessity of isCGM than males—a motivational advantage that correlated directly with better adherence [12]—indicating that female patients’ stronger sense of device necessity represents a psychosocial asset that clinicians can leverage to support consistent monitoring engagement.

Technology burden and body image concerns emerged as structurally gendered barriers to diabetes device use with clear clinical consequences. Tanenbaum et al. identified body image concerns, device visibility, and interference with clothing and intimacy as pump-related barriers disproportionately endorsed by females (*p* < 0.01), while males more commonly cited device complexity as a CGM barrier [5]—findings that reveal not merely a quantitative difference in burden level but a qualitative difference in the nature of barriers experienced, with female patients navigating psychosocial and aesthetic challenges that standard clinical consultations often fail to address. Fabris et al. added a hormonal dimension, documenting increased frustration with technology alerts during the luteal phase [3], indicating that cyclical hormonal changes may amplify perceived technology burden in females and contribute to the higher psychosocial distress documented in Section 3.3.3. Clinicians should therefore adopt gender-sensitive device counselling frameworks that explicitly address body image, social visibility, and hormonal interactions with device use.

#### 3.3.4. Technology-Specific Findings

AHCL systems showed the greatest promise among available technologies for reducing gender-related disparities in diabetes outcomes, acting through multiple clinically relevant mechanisms simultaneously. The TIR gender gap was smaller under AHCL than under conventional CSII [13], demonstrating that closed-loop automation can narrow glycaemic outcome inequity without requiring additional behavioural effort from patients. Automated management of menstrual cycle-related hormonal variability substantially reduced the cyclical TIR fluctuations that burden females on conventional therapy [3], directly addressing the most clinically impactful female-specific physiological challenge identified in this review. The MiniMed 780G system provided particular benefit to female users through its adaptive hormonal adjustment management capabilities [2], while AHCL systems as a class reduced overall management burden across both genders [4]—a feature likely to be disproportionately valued by female patients who already carry higher psychosocial distress loads. Taken together, these findings support clinical prioritisation of AHCL in female patients, particularly those with evidence of cycle-related glycaemic instability or elevated technology burden.

Digital health interventions demonstrated a consistent female engagement advantage with direct clinical implications for technology prescription strategies. Higher female completion rates and greater HbA1c benefit among programme completers [8,14] indicate that remote digital platforms are not merely adjunctive tools but may represent preferential therapeutic modalities for female patients—particularly those who have discontinued or are at risk of discontinuing insulin pump therapy. Clinicians should consider proactive prescription of digital health programmes as a core component of a gender-sensitive diabetes technology strategy, recognising that the female advantage in engagement and glycaemic outcomes extends the potential of technology-supported care well beyond device-based approaches.

## 4. Discussion

### 4.1. Summary of Findings

**Figure 2:**
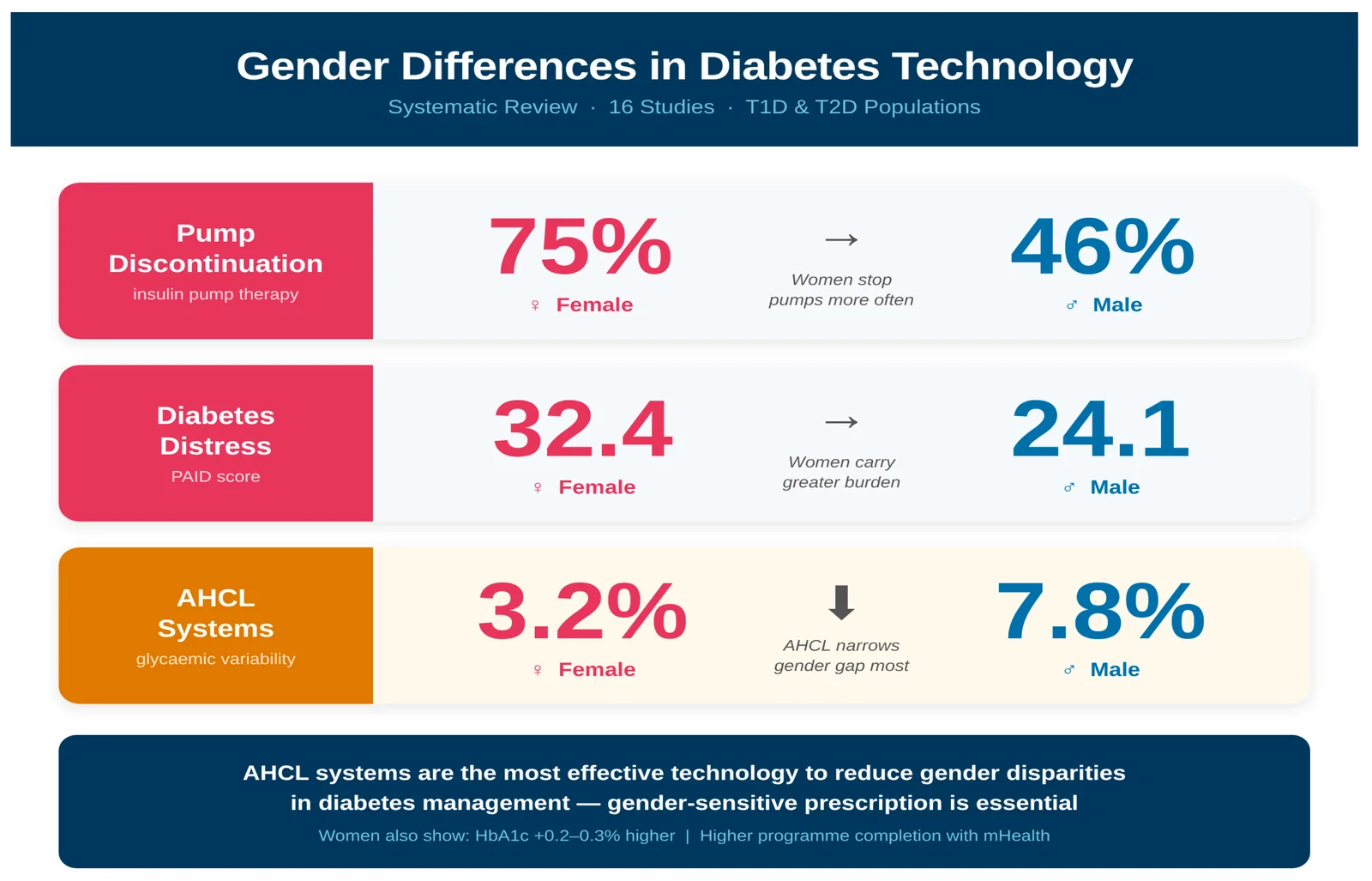
Graphical summary of gender differences in diabetes technology adherence and outcomes across three primary domains (adherence, glycaemic outcomes, and psychosocial impact), providing a visual overview of the five key findings described in the text. AHCL = advanced hybrid closed-loop; CGM = continuous glucose monitoring; isCGM = intermittently scanned CGM; PAID = Problem Areas in Diabetes scale; TIR = time-in-range. Note: insulin pump discontinuation percentages derive primarily from a single predominantly paediatric cohort [1] and should be interpreted with caution when extrapolating to adult populations. This is an original figure created by the authors; no external copyright applies.

This systematic review identified five principal gender-related patterns in diabetes technology outcomes (Key Findings 1–5 below; Figure 2 provides a graphical overview of three primary domains).

**Key Finding 1: Gender-specific adherence varies by technology type.** Females showed higher insulin pump discontinuation driven by body image concerns—a directional finding consistent across three studies but deriving principally from a single predominantly paediatric cohort [1,5,9,10], while males showed lower isCGM scan frequency linked to perceived necessity (single small cohort, n = 72 [12]; independent replication needed). Contrasting patterns by technology type indicate that different devices pose distinct gender-specific barriers.

**Key Finding 2: Glycaemic control differences are modest but consistent.** HbA1c differences (0.2–0.3%) and TIR patterns were modest yet consistent across studies [6,9,13]. Menstrual cycle physiology imposed clinically relevant glycaemic variability in females, substantially mitigated by AHCL [3]. The perception-reality gap—females rating control more negatively than warranted by objective data—merits clinical attention [6].

**Key Finding 3: Psychosocial disparities are substantial.** Females consistently report higher distress, greater fear of hypoglycaemia, and stronger body image concerns [5,11], persisting after adjustment for glycaemic control, indicating independent psychological contributions [11].

**Key Finding 4: AHCL systems reduce gender disparities.** Automation addresses female-specific challenges—hormonal variability, management burden, and cycle-related glycaemic instability—while maintaining high satisfaction across genders [2,3,4,13,15].

**Key Finding 5: Digital health shows higher female engagement.** Remote monitoring programmes achieve higher completion rates and greater HbA1c reductions in females [8,14].

Beyond biological sex differences, sociocultural determinants play a pivotal role in shaping technology acceptance. Gender-specific body image norms, differential healthcare engagement patterns, socioeconomic barriers including insurance coverage disparities, cultural attitudes toward technology, and caregiving roles further modulate the interface between sex/gender and diabetes technology outcomes. Future research should explicitly measure sociocultural variables—including socioeconomic status, cultural background, and caregiving burden—as potential mediators of sex/gender effects on technology adherence.

These five findings are broadly consistent with, yet substantially extend, prior narrative reviews [2,4,14]. Whereas earlier work noted isolated sex differences in pump use or HbA1c, this review demonstrates that gender effects are technology-specific and multidimensional: the same female patients who are more likely to discontinue insulin pumps are simultaneously more likely to complete remote monitoring programmes and report higher perceived necessity of CGM. This “adherence paradox” underscores that gender influences not merely the quantity but the pattern of technology engagement, shaped by the unique interface between device characteristics and gender-specific psychosocial determinants. Clinicians and policymakers must therefore avoid monolithic generalisations about gender and technology adherence, instead adopting a nuanced, technology-specific approach to gender-sensitive care.

A key clinical question arising from the adherence paradox is whether the predominance of female patients in pump discontinuation groups—reported by De Vries et al. [1] in a single predominantly paediatric cohort—should prompt clinicians to restrict pump prescribing in female patients. The available evidence does not support this conclusion. The 75% and 46% figures represent the proportions of females within the discontinuation and continuation groups, respectively, not absolute per-sex discontinuation rates; in the absence of absolute rates from large, adult, prospective cohorts, policy changes to prescribing eligibility would be premature. The consistent directional finding across Gandhi et al. [10] and Boettcher et al. [9] warrants vigilance regarding pump persistence in female patients, but the appropriate response is proactive barrier identification and management—including body image counselling, patch-pump prescription, and peer support—rather than restriction of access to an effective therapy.

An additional dimension meriting consideration is gender differences in the initial uptake and prescription of diabetes technologies. The barriers identified in this review—body image concerns, device complexity, perceived necessity—may manifest differently at the point of technology initiation versus sustained use; future research should examine whether sex-based differences in clinician prescribing contribute to differential uptake rates.

**Clinical implications.** The central clinical question raised by these findings is whether the observed higher insulin pump discontinuation among female patients should lead to differential prescribing. The answer is no: the evidence does not support withholding pump therapy from female patients. Instead, clinicians should implement proactive barrier management before and during device initiation, specifically: offering patch-pump alternatives for patients with device-visibility concerns; providing targeted body image counselling at the pre-prescription stage; and establishing structured peer-support with experienced female pump users. Clinicians should: (i) assess gender-specific barriers before prescribing technology; (ii) provide targeted education addressing body image concerns in females and technical complexity in males; (iii) discuss menstrual cycle effects and consider AHCL in females with cycle-related glycaemic variability; (iv) interpret female patients’ self-reported poor control in the context of the perception-reality gap; (v) consider digital health interventions particularly for female patients; and (vi) advocate for equitable access to AHCL systems. Beyond individual clinical encounters, these findings carry implications for health systems and technology developers. Reimbursement policies should account for differential cost-effectiveness of AHCL systems in female patients with hormonally driven glycaemic variability. User interface design teams should engage gender-diverse focus groups to reduce device-related body image barriers. Healthcare providers should be trained to distinguish objective glycaemic metrics from subjective distress, acknowledging that the perception-reality gap may represent a valid psychosocial burden warranting specific intervention. Structured peer-support programmes and gender-specific education modules could complement technology prescription to address the psychosocial determinants of adherence identified across multiple included studies [5,11].

### 4.2. Limitations

This review has several important limitations. Methodological heterogeneity across included studies precluded meta-analysis. Operational definitions of adherence varied substantially. Most cohorts were drawn from predominantly white European or North American populations, limiting generalisability. Adequate adjustment for confounders (socioeconomic status, healthcare access, diabetes duration) was lacking in many studies. Follow-up periods were often short (≤6 months). Publication bias may inflate apparent effect sizes. The interchangeable use of “gender” and “sex” terminology in source studies limits mechanistic interpretation, and no included study examined diabetes technology outcomes in transgender or non-binary individuals—a critical gap for future research. Additionally, the interplay between biological sex and psychosocial gender is rarely disentangled in the included literature. Future studies should prospectively collect both sex-assigned-at-birth and self-identified gender data to enable nuanced analysis. The absence of long-term follow-up data (most studies ≤ 6 months) further limits understanding of whether initial gender differences in adherence attenuate or persist over time, and whether AHCL-mediated reductions in gender disparities are durable across multiple menstrual cycles and seasons of life. Additionally, several methodological limitations specific to this review’s design warrant explicit acknowledgement. First, the study was deposited on Zenodo as a document repository record and was not registered in a prospective systematic review registry (e.g., PROSPERO, OSF, INPLASY); formal prospective registration was not performed. Second, the bibliographic search was limited to SciSpace, Google Scholar, and PubMed; major databases including Embase, Scopus, Web of Science, CINAHL, and the Cochrane Library were not searched, which may have resulted in incomplete retrieval and increased the risk of publication bias. Third, the relevance scoring system (0–5) was developed by the review team without independent external validation; while it was applied consistently, the absence of a peer-reviewed rubric is an acknowledged limitation. Fourth, data extraction was confined to the longest available follow-up for each study; this approach may fail to capture early-phase differences that subsequently attenuate, and baseline-to-endpoint changes cannot be assessed from this design. Fifth, this review included systematic reviews and narrative reviews as source studies; although these were treated as contextualisation sources only, the potential for indirect evidence duplication cannot be entirely excluded. Furthermore, none of the more recently published studies included in this review (2025–2026) explicitly assessed self-identified gender identity (including queer, non-binary, or gender-diverse identification) in their study design, confirming this as a persistent gap even in contemporary literature.

### 4.3. Future Research Directions

Several priority areas for future research emerge from this review. First, randomised controlled trials specifically designed to test gender-tailored technology interventions are urgently needed, with gender-stratified primary endpoints, validated patient-reported outcome measures, and active follow-up of at least 12 months. Second, studies should explicitly measure both sex-assigned-at-birth and self-reported gender identity to disentangle biological and psychosocial contributions and to include transgender and non-binary individuals—currently absent from the literature.

Third, mechanistic research should clarify the interplay between sex hormones—particularly oestrogen and progesterone cycles—and insulin sensitivity parameters captured by AHCL algorithms, with the goal of developing adaptive closed-loop protocols that anticipate menstrual-phase glucose dynamics. Fourth, health economic evaluations should model gender-stratified cost-effectiveness of AHCL versus CSII to inform equitable reimbursement decisions. Fifth, qualitative and mixed-methods research can complement quantitative approaches by capturing lived experiences of technology burden and body image in diverse cultural and socioeconomic contexts. Together, these research directions would substantially advance gender-sensitive diabetes technology practice.

## 5. Conclusions

Sex-based and gender-related differences—from limited and heterogeneous evidence—are apparent in diabetes technology adherence, glycaemic outcomes, and psychosocial experiences. Based on limited and predominantly paediatric evidence, females appear to face higher insulin pump discontinuation rates (based on group sex compositions in a single predominantly paediatric cohort, not absolute sex-stratified discontinuation rates) but engage more consistently with isCGM and digital health programmes; males demonstrate lower isCGM scan frequency—a finding from a single small cohort (Sousa et al., n = 72) requiring independent replication. Gender differences in HbA1c are modest (0.2–0.3%) but consistent; females exhibit lower glycaemic variability despite slightly lower TIR, and menstrual cycle physiology imposes additional glycaemic challenges partially mitigated by AHCL systems. Females consistently report higher distress and more negative perceptions of glycaemic control, even when objective metrics are comparable to or better than males. This review—encompassing 16 studies, over 52,000 patients, and four technology categories—provides the most comprehensive synthesis to date of gender-specific patterns in diabetes device use.

AHCL systems represent the most promising current technology for reducing gender disparities by automating insulin delivery and addressing hormonal variability burden. Digital health interventions show particular effectiveness for female engagement.

These findings support the implementation of gender-sensitive approaches in diabetes technology prescription, education, and clinical support. Future research should investigate the underlying mechanisms, assess outcomes in transgender and non-binary populations, and test gender-tailored interventions in randomised controlled trials. The evidence synthesised here provides an actionable foundation for clinicians, technology developers, health economists, and policymakers. Female patients may benefit most from early escalation to AHCL systems and structured psychosocial support addressing diabetes distress and the perception-reality gap in glycaemic control, while male patients may require targeted education to promote consistent CGM scan frequency. Embedding these gender-specific considerations into clinical protocols, device design, and reimbursement frameworks represents a concrete next step toward minimising technology-related health disparities in diabetes care.

## Data Availability

The data presented in this study are available in the references cited in this article. This systematic review is deposited as a public document repository record on Zenodo (CERN): https://doi.org/10.5281/zenodo.21257084 (accessed on 8 July 2025). Note: Formal prospective registration in a dedicated systematic review registry (e.g., PROSPERO, OSF, INPLASY) was not performed prior to the commencement of this review, as the systematic search and data extraction were initiated before the protocol was formally registered. Recognising this limitation, the studysma protocol was retrospectively deposited on Zenodo (CERN; DOI: 10.5281/zenodo.21257084) as a document repository record to ensure transparency. The authors acknowledge that Zenodo is a document repository and is not equivalent to a prospective systematic review registry and recognise the absence of prospective registration as a methodological limitation of the present review.

## Supplementary Materials

The following supporting information can be downloaded at: https://www.mdpi.com/article/10.3390/jcm15176740/s1, PRISMA 2020 Checkilist. Reference [7] is cited in Supplementary Materials.

## Abbreviations

The following abbreviations are used in this manuscript:

AHCL	Advanced hybrid closed-loop
CGM	Continuous glucose monitoring
CSII	Continuous subcutaneous insulin infusion
CV	Coefficient of variation
DTSQ	Diabetes Treatbriesment Satisfaction Questionnaire
HbA1c	Glycated haemoglobin
isCGM	Intermittently scanned continuous glucose monitor
mHealth	Mobile health
PAID	Problem Areas in Diabetes scale
PRISMA	Preferred Reporting Items for Systematic Reviews and Meta-Analyses
T1D	Type 1 diabetes mellitus
T2D	Type 2 diabetes mellitus
TAR	Time above range
TBR	Time below range
TIR	Time-in-range
