# Supplementary material for: Gender Differences in Diabetes Technology: Adherence and Outcomes—A Systematic Review
Source: J Clin Med. 2026 Aug 30;15(17):6740. doi: 10.3390/jcm15176740 (PMC13565765; doi:10.3390/jcm15176740)
Supplement: Supplementary file 1 [file jcm-15-06740-s001.zip › jcm-4490901-supplementary.pdf]

PRISMA 2020 Checklist

Section and Topic	Item #	Checklist item	Location where item is reported
**TITLE**			
Title	1	Identify the report as a systematic review.	Title page — running head ('Systematic Review') and title both identify the document as a systematic review
**ABSTRACT**			
Abstract	2	See the PRISMA 2020 for Abstracts checklist.	Abstract section
**INTRODUCTION**			
Rationale	3	Describe the rationale for the review in the context of existing knowledge.	Section 1.1 (Introduction: Rationale)
Objectives	4	Provide an explicit statement of the objective(s) or question(s) the review addresses.	Section 1.2 (Introduction: Objectives)
**METHODS**			
Eligibility criteria	5	Specify the inclusion and exclusion criteria for the review and how studies were grouped for the syntheses.	Section 2.2 (Methods: Eligibility Criteria)
Information sources	6	Specify all databases, registers, websites, organisations, reference lists and other sources searched or consulted to identify studies. Specify the date when each source was last searched or consulted.	Section 2.1 (Methods: Search Strategy) — SciSpace, SciSpace Full Text, Google Scholar, PubMed; 11 queries; searches conducted up to July 2025
Search strategy	7	Present the full search strategies for all databases, registers and websites, including any filters and limits used.	Section 2.1 (Methods: Search Strategy) — full Boolean search terms and example PubMed query provided
Selection process	8	Specify the methods used to decide whether a study met the inclusion criteria of the review, including how many reviewers screened each record and each report retrieved, whether they worked independently, and if applicable, details of automation tools used in the process.	Section 2.3 (Methods: Screening Process) — two independent reviewers; two-stage process; Cohen's kappa reported
Data collection process	9	Specify the methods used to collect data from reports, including how many reviewers collected data from each report, whether they worked independently, any processes for obtaining or confirming data from study investigators, and if applicable, details of automation tools used in the process.	Section 2.4 (Methods: Data Extraction) — standardised form; two independent reviewers; discrepancies resolved by discussion
Data items	10a	List and define all outcomes for which data were sought. Specify whether all results that were compatible with each outcome domain in each study were sought (e.g. for all measures, time points, analyses), and if not, the methods used to decide which results to collect.	Section 2.4 (Methods: Data Extraction) — adherence, glycaemic control (HbA1c, TIR, TAR, TBR, variability), and patient-reported outcomes listed
	10b	List and define all other variables for which data were sought (e.g. participant and intervention characteristics, funding sources). Describe any assumptions made about any missing or unclear information.	Section 2.4 (Methods: Data Extraction) — study design, diabetes technology type, participant characteristics, funding
Study risk of bias assessment	11	Specify the methods used to assess risk of bias in the included studies, including details of the tool(s) used, how many reviewers assessed each study and whether they worked independently, and if applicable, details of automation tools used in the process.	Section 2.5 (Methods: Quality Assessment and Risk of Bias) — Newcastle-Ottawa Scale (NOS) for cohort/cross-sectional studies; AMSTAR-2 for systematic reviews; two independent reviewers
Effect measures	12	Specify for each outcome the effect measure(s) (e.g. risk ratio, mean difference) used in the synthesis or presentation of results.	Not applicable — qualitative narrative synthesis only; no meta-analysis performed (Section 2.5)

PRISMA 2020 Checklist

Section and Topic	Item #	Checklist item	Location where item is reported
Synthesis methods	13a	Describe the processes used to decide which studies were eligible for each synthesis (e.g. tabulating the study intervention characteristics and comparing against the planned groups for each synthesis (item #5)).	Section 2.5 (Methods: Quality Assessment) — studies eligible for qualitative synthesis based on clinical and methodological heterogeneity assessment
	13b	Describe any methods required to prepare the data for presentation or synthesis, such as handling of missing summary statistics, or data conversions.	Section 2.4 (Methods: Data Extraction) — longest follow-up extracted; assumptions for missing data described
	13c	Describe any methods used to tabulate or visually display results of individual studies and syntheses.	Table 1 (study characteristics); Figure 1 (PRISMA 2020 flow diagram); Figure 2 (graphical summary of gender differences)
	13d	Describe any methods used to synthesize results and provide a rationale for the choice(s). If meta-analysis was performed, describe the model(s), method(s) to identify the presence and extent of statistical heterogeneity, and software package(s) used.	Section 3.3 (Results: Synthesis of Evidence) — qualitative narrative synthesis; meta-analysis not performed due to heterogeneity
	13e	Describe any methods used to explore possible causes of heterogeneity among study results (e.g. subgroup analysis, meta-regression).	Not formally assessed; clinical and methodological heterogeneity precluded meta-analysis; acknowledged in Section 2.5 and Section 4.2 (Limitations)
	13f	Describe any sensitivity analyses conducted to assess robustness of the synthesized results.	Not performed — qualitative synthesis only; acknowledged in Section 4.2 (Limitations)
Reporting bias assessment	14	Describe any methods used to assess risk of bias due to missing results in a synthesis (arising from reporting biases).	Not formally assessed; publication bias acknowledged as a limitation in Section 4.2
Certainty assessment	15	Describe any methods used to assess certainty (or confidence) in the body of evidence for an outcome.	Section 2.5 (NOS quality ratings: high ≥7 stars, moderate 4–6, low <4); 11/16 studies (69%) rated moderate-to-high quality; formal GRADE not applied
**RESULTS**			
Study selection	16a	Describe the results of the search and selection process, from the number of records identified in the search to the number of studies included in the review, ideally using a flow diagram.	Section 3.1 (Results: Study Selection); Figure 1 (PRISMA 2020 flow diagram)
	16b	Cite studies that might appear to meet the inclusion criteria, but which were excluded, and explain why they were excluded.	Section 2.3 (Methods: Screening Process) — primary exclusion reasons: lack of gender comparison (57%), non-technology focus (24%), single-gender population (15%); 4 full-text exclusions explained
Study characteristics	17	Cite each included study and present its characteristics.	Section 3.2 (Results: Study Characteristics); Table 1
Risk of bias in	18	Present assessments of risk of bias for each included study.	Section 2.5 (Methods: Quality Assessment) — NOS ratings reported;

Section and Topic	Item #	Checklist item	Location where item is reported
studies			11/16 studies moderate-to-high quality
Results of individual studies	19	For all outcomes, present, for each study: (a) summary statistics for each group (where appropriate) and (b) an effect estimate and its precision (e.g. confidence/credible interval), ideally using structured tables or plots.	Section 3.3 (Results: Synthesis of Evidence) — individual study results presented with summary statistics and p-values
Results of syntheses	20a	For each synthesis, briefly summarise the characteristics and risk of bias among contributing studies.	Section 3.3 (Results: Synthesis of Evidence) — evidence synthesised by technology type and outcome domain
	20b	Present results of all statistical syntheses conducted. If meta-analysis was done, present for each the summary estimate and its precision (e.g. confidence/credible interval) and measures of statistical heterogeneity. If comparing groups, describe the direction of the effect.	Not applicable — no meta-analysis performed; qualitative narrative synthesis only (Section 2.5)
	20c	Present results of all investigations of possible causes of heterogeneity among study results.	Not formally assessed; Section 4.2 (Limitations) acknowledges heterogeneity across designs, populations, and definitions
	20d	Present results of all sensitivity analyses conducted to assess the robustness of the synthesized results.	Not performed; Section 4.2 (Limitations)
Reporting biases	21	Present assessments of risk of bias due to missing results (arising from reporting biases) for each synthesis assessed.	Not formally assessed; publication bias acknowledged as a limitation (Section 4.2)
Certainty of evidence	22	Present assessments of certainty (or confidence) in the body of evidence for each outcome assessed.	Section 2.5 (NOS quality ratings); formal GRADE certainty assessment not applied
**DISCUSSION**			
Discussion	23a	Provide a general interpretation of the results in the context of other evidence.	Section 4.1 (Discussion: Summary of Findings) — five key findings interpreted in context of existing evidence
	23b	Discuss any limitations of the evidence included in the review.	Section 4.2 (Discussion: Limitations) — evidence-level limitations described (heterogeneity of definitions, short follow-up, limited confounding adjustment, generalisability)
	23c	Discuss any limitations of the review processes used.	Section 4.2 (Discussion: Limitations) — review-level limitations described (no prospective registration; restricted database coverage; unvalidated scoring rubric; longest-follow-up extraction only; inclusion of narrative/systematic reviews)
	23d	Discuss implications of the results for practice, policy, and future research.	Section 4.1 (clinical implications — gender-sensitive prescribing, body image counselling, AHCL prioritisation); Section 4.3 (Future Research Directions — RCTs, transgender populations, mechanistic research, health economics)

PRISMA 2020 Checklist

Section and Topic	Item #	Checklist item	Location where item is reported
**OTHER INFORMATION**			
Registration and protocol	24a	Provide registration information for the review, including register name and registration number, or state that the review was not registered.	Section 2.1 (Methods: Search Strategy); Study Registration section — retrospective Zenodo deposit (DOI: 10.5281/zenodo.21257084); not prospectively registered in PROSPERO, OSF, or INPLASY; acknowledged as a limitation
	24b	Indicate where the review protocol can be accessed, or state that a protocol was not prepared.	Section 2.1; Study Registration section — Zenodo deposit (DOI: 10.5281/zenodo.21257084) serves as repository record; no separate protocol document was prepared prospectively
	24c	Describe and explain any amendments to information provided at registration or in the protocol.	Not applicable — no prospective registration; no amendments to report
Support	25	Describe sources of financial or non-financial support for the review, and the role of the funders or sponsors in the review.	Funding section — 'This research received no external funding'
Competing interests	26	Declare any competing interests of review authors.	Conflicts of Interest section — 'The authors declare no conflicts of interest'
Availability of data, code and other materials	27	Report which of the following are publicly available and where they can be found: template data collection forms; data extracted from included studies; data used for all analyses; analytic code; any other materials used in the review.	Data Availability Statement; Zenodo public repository: https://doi.org/10.5281/zenodo.21257084

From: Page MJ, McKenzie JE, Bossuyt PM, Boutron I, Hoffmann TC, Mulrow CD, et al. The PRISMA 2020 statement: an updated guideline for reporting systematic reviews. BMJ 2021;372:n71. doi: 10.1136/bmj.n71.
This work is licensed under CC BY 4.0. To view a copy of this license, visit https://creativecommons.org/licenses/by/4.0/
